# Assessing sugar-sweetened beverage consumption in early pregnancy using a substance abuse framework

**DOI:** 10.1038/s41598-023-46265-y

**Published:** 2023-11-03

**Authors:** Chin-Ru Ker, Hao-Ching Yang, Shih-Han Wang, Te-Fu Chan

**Affiliations:** 1 Graduate Institute of Clinical Medicine, College of Medicine, Kaohsiung Medical University, Kaohsiung, 80708 Taiwan; 2 Department of Obstetrics and Gynecology, Kaohsiung Medical University Hospital, Kaohsiung Medical University, 100 Tzyou 1st Road, Kaohsiung, 80708 Taiwan; 3 Center of Cancer Research, Kaohsiung Medical University, Kaohsiung, 80708 Taiwan

**Keywords:** Addiction, Human behaviour, Disease prevention, Nutrition, Public health, Weight management

## Abstract

Sugar-sweetened beverages (SSB) are previously reported to jeopardize maternal fetal health, most well-known for gestational diabetes, childhood obesity, and cognitive impairment. Although warnings and diet recommendations urge pregnant women to consume less SSB, there is no noticeable difference in their behavior. How and why reproductive women change their SSB consumption patterns were not investigated previously. Our study aims to investigate beverage consumption patterns and how these patterns change in pregnancy in the context of substance use disorder (SUD). We invited all pregnant women visiting the clinic to answer a structured 20-min questionnaire every trimester during the regular antennal visit. At the end of the study, 337 pregnant women aged over 20 participated. A total of 301 responses entered for final analysis, with a response rate of 89.3%. Our finding showed those with high DSM-5-TR scores reduced SSB intake after becoming pregnant, while those with mild or low DSM-5-TR scores increased SSB intake after becoming pregnant. The top 3 factors related to their SSB consumption were “use despite of known health hazard (n = 133)”, “increased desire to drink (n = 88)”, and “excessive time spent on seeking SSB (n = 85)”. The least reported factors were in the domains of social impairment (ranging from n = 3 to n = 26), pharmacologic effects (i.e., tolerance (n = 24) and withdrawal (n = 70). When participants reduced SSB consumption after becoming pregnant, their choice of beverages largely shifted to sugarless beverage but not much plain water. The result provided new insights in deciphering pregnant women’s psychomotor factors for SSB intake, which served as useful references for making clinical or even public health recommendations.

## Introduction

Sugar-sweetened beverages (SSB) are drinks with added sugar that lead to weight gain but no nutritional value. A growing body of evidence supports adverse maternal and neonatal outcomes associated with excessive sugar intake during pregnancy^1^. Gestational diabetes^2,3^, preeclampsia^3,4^, preterm delivery^5,6^, childhood obesity^3,7,8^, congenital heart defect^9^, pediatric asthma^10^, impaired cognitive development in children^11^ are some consequences discussed in the published literature. Dietary recommendations from official authorities that urge for cautious and restricted added sugars in pregnancy are emerging^12–14^. Despite of these concerning messages, excessive use of sugars is still observed in epidemiology studies globally^15–19^. According Dietary Guidelines for Americans 2020–2025, 70% pregnant women and 51% lactating women consumed more sugars than the recommended intake (10% of total energy)^20^. The difficulty of curtailing SSB consumption raised speculations of its addictive potential or its role as a coping strategy to emotional distress faced by pregnant women^21^.

**Figure 1:**
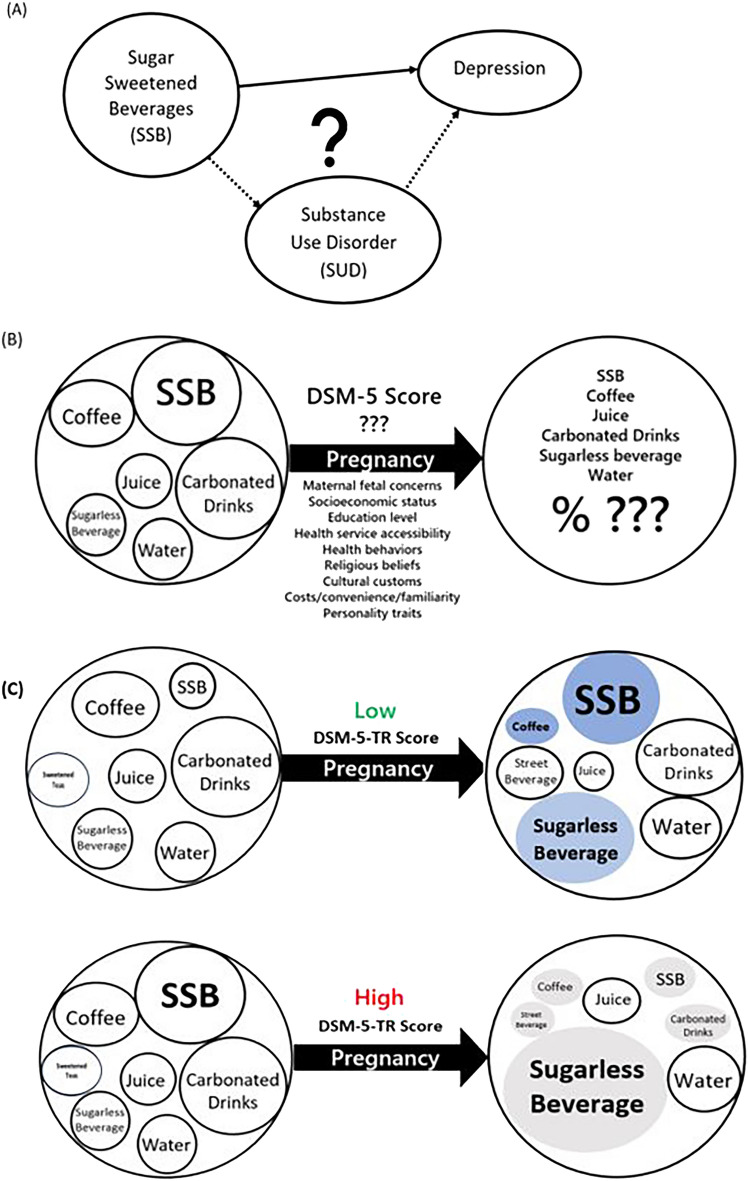
Study question and proposed construct of beverage consumption changes in early pregnancy. (**A**) The possible relationship between SSBs consumption, substance use disorder and depression. (**B**) The proposed framework to study pre- and post-pregnancy SSB consumption pattern changes. (**C**) Differential SSB consumption during early pregnancy according to DSM-5 scores; filled circles denotes changes with statistical significance.

Substance use disorder (SUD) is an illness of prolonged, repeated use of a particular substance with a high dose or frequency. The substances are conventionally defined as psychoactive compounds including nicotine, alcohol, cannabinoids, opioids, depressants, stimulants and hallucinogens, with the potential to cause health and social problems^22^. More and more reports relate the addiction-like symptoms to eating disorders, such as strong cravings and compulsive overeating, using the same diagnostic criteria^23^. SSB stood out as one such item, with many studies demonstrating drug-like effects, namely bingeing, craving, tolerance and withdrawal in animal studies, brain neurochemistry and behavioral studies in the adolescent and general population^24,25^. Few, however, have examined SSB consumption in pregnant women in light of substance use. Many people with SUD experience other mental disorders. In the case of pregnant women, a growing number of studies have found that increased SSB consumption places them at higher risk of postpartum depression (PPD)^21,26^. This validates the experimentation of characterizing SSB as an SUD, for findings could help us make inferences about how it might affect PPD (Fig. 1A). In turn, we could design better prevention strategies for these demographics.

In search of a tool in assessing the degree and various facets of SSB desire, the diagnostic criteria for substance use disorder (SUD) defined by the fifth edition of Diagnostic and Statistical Manual, text revision, (DSM-5-TR) stood out as a suitable candidate. The criteria comprised of 11 items that evaluate comprehensively the various domains in addictive behaviors, including impaired control, hazardous use, social impairment, and pharmacologic effects (withdrawal and tolerance). Its use has a long history with wide applications in the medical, social, economic, and cultural settings. It is flexible and could be readily customized for any substances or even non-substance behaviors in the research setting outside the scope defined in DSM-5-TR^27–31^, including betelnuts, gambling, UV light tanning, etc. Others utilize it as a unidimensional continuum such that scores from each diagnostic category add up to a total score to indicate level of obsession severity^32^.

Fluid intake in general daily lives is quite consistent. The choices of what to drink become important, for unhealthy drinks could compete and replace what could have been healthier choices. It is known that pregnant women modify their beverage choices during the transition, most noticeably with reduced alcohol, caffeine, SSB and increased milk, juice, and vitamins^33,34^. Evidence was found in Chinese, Malaysian, Indian and Norway populations^33,35^. Lundeen et al. specifically pointed out that 1 in 4 non-pregnant women versus 1 in 5 pregnant women consumed SSBs at least once per day^36^. Some contributing factors to the change include altered olfaction and tastes, hormone fluctuations, personality traits, cultural beliefs, but mostly maternal fetal health concerns^37–39^. Others found correlations with socioeconomic status, race, stress, body mass index and education levels, although results were inconsistent^6,15,33,36,40–42^. Whether or not the attitude for SSB also plays a role in pregnant women’s beverage selection process is a topic of interest (Fig. 1B). The current study aims to characterize pregnant women’s beverage drinking patterns in terms of substance abuse symptoms, categorized by four domains in eleven items as laid out in the modified DSM-5-TR SUD criteria for SSB. The results will serve as a starting point to depict SSBs consumption patterns in pregnant women and to decipher the possible underlying motives, so that awareness could be raised in pregnant women, in care providers and even healthcare policy makers to regulate SSB intake and to enhance maternal fetal health accordingly.

## Results

**Figure 2:**
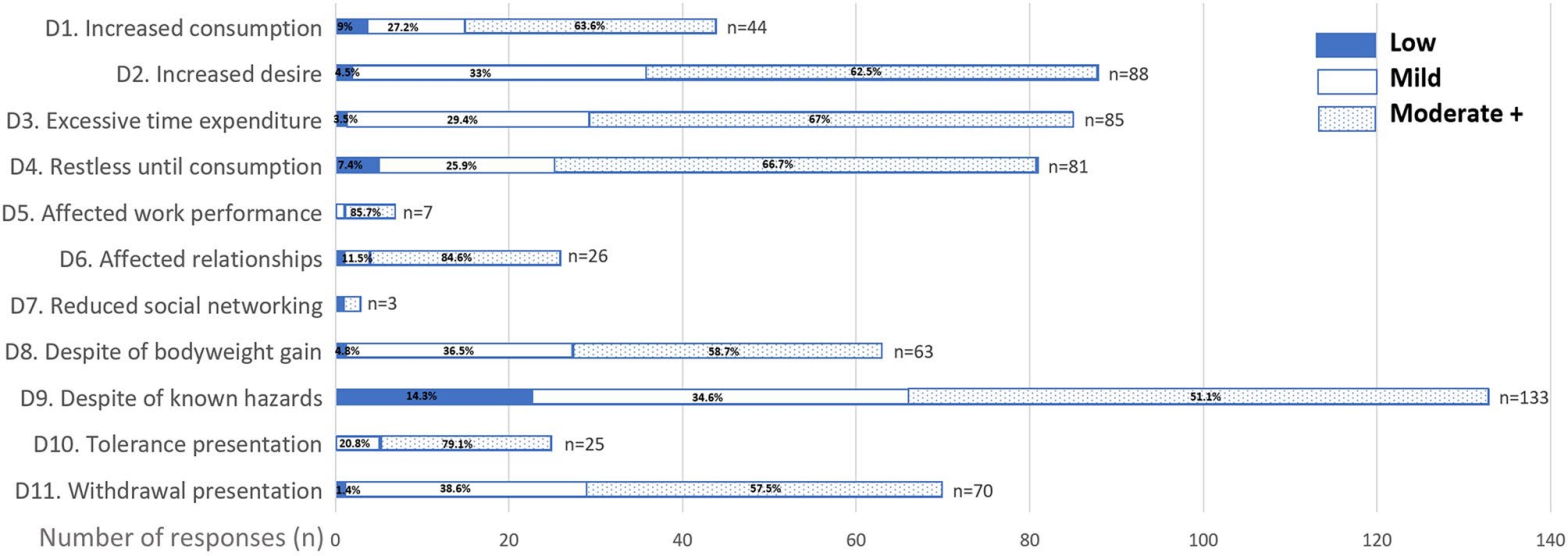
Participants’ responses sorted by degree of beverage consumption scores (low, mild, moderate+) and specific DSM-5-TR questions (D1–D11). Numbers are given in number (n) and percentage (%).

The single-centered observational study cohort is composed of 301 participants with mean age of 33.7 ± 4.5 years and body mass index of 23.2 ± 4.3 mg/m^2^. Forty-eight point two percent of these women are primiparous and 87.4% had college or higher education level. These variables were comparable among low, mild, and moderate DSM-5-TR score groups (Table 1). Participants’ attitudes and use patterns for beverages prior to pregnancy were assessed with results presented in Fig. 2. All 11 questions received increasing positive responses with increasing scores: low (0.0–12.5%), mild (0.0–60.5%) and moderate+ (2.7–93.2%) (Supplementary Table 1). The top 3 questions responded were using despite of known hazards to health (n = 133), increased desire to drink (n = 88) and excessive time spent on seeking these drinks (n = 85). The least responses were for reduced social networking (n = 3), affected work performance (n = 7) and tolerance symptoms towards these drinks (n = 24).

**Table 1:** Demographic distribution of participants, sorted by modified DSM-5-TR scores for beverages.

Variables	Modified DSM-5-TR scores for Beverages			Total
	Low	Mild	Moderate+	
Number	152	76	73	301
Age (years old)	33.9 ± 4.5	33.7 ± 4.8	33.4 ± 4.2	33.7 ± 4.5
Education level (n)				
High school	19 (12.5%)	10 (13.1%)	9 (12.3%)	38 (12.6%)
College	115 (75.7%)	49 (64.5%)	55 (75.3%)	219 (72.8%)
Graduate school	18 (11.8%)	17 (22.3%)	9 (12.3%)	44 (14.6%)
Weight (kilograms)	58.5 ± 11.1	61.4 ± 12.1	61.0 ± 12.7	59.8 ± 11.8
Height (centimeters)	159.8 ± 4.9	161.2 ± 5.5	161.3 ± 5.7	160.5 ± 5.3
Body mass index (kg/m^2^)^#^	22.8 ± 4.0	23.6 ± 4.4	23.4 ± 4.7	23.2 ± 4.3
Nulliparity (n)	64 (42.1%)	39 (51.3%)	42 (57.5%)	145 (48.2%)

Data are given in number (n), percentage (%) or mean ± standard deviations (mean ± SD).

^#^Denotes BMI at study entry.

Beverage preferences among the participants before and after pregnancy were reported in Table 2. Before pregnancy, higher DSM-5-TR scores correlate with increased SSB consumption (p = 0.027 for low versus mild group, p < 0.001 for mild versus moderate + group and p < 0.001 for low versus moderate + group comparisons). Higher DSM-5-TR scores also correlate with more frequent sweetened teas consumption (p = 0.029, p < 0.001, p < 0.001, respectively), and decreased plain water intake (p = 0.648, p = 0.031, p < 0.001, respectively). In early pregnancy, higher DSM-5-TR scores correlate with increased consumption in sugarless beverages (p = 1.000 for low versus mild group, p = 0.036 for mild versus moderate + group, and p = 0.012 for low versus moderate + group comparisons). Higher DSM-5-TR scores were also associated with more frequent sweetened teas consumption (p = 0.043, p = 0.048, p < 0.01, respectively) and decreased plain water intake (p = 0.888, p = 0.004, p < 0.01, respectively).

**Table 2:** Beverage consumption pattern analysis by timing of assessment (pre- and early pregnancy) and different degree of DSM-5-TR score groups (low, mild and moderate+).

Variables	Time of assessment	Modified DSM-5-TR Scores for beverages (mean ± SD)			One-way ANOVA		
		Low (points 0–1)	Mild (points 2–3)	Moderate+ (points ≥ 4)	p-value*	p-value^	p-value^@^
SSB^&^	Pre-pregnancy	314.14 ± 272.19	424.34 ± 305.56	616.44 ± 312.03	0.027	< 0.001	< 0.001
	Early pregnancy	412.83 ± 312.89	424.34 ± 316.28	417.81 ± 322.90	0.967	0.992	0.994
	Paired t test/p-value^#^	0.003	1.000	< 0.001	–		
Sugarless beverage**	Pre-pregnancy	633.22 ± 635.54	687.50 ± 703.71	767.12 ± 688.86	0.845	0.767	0.371
	Early pregnancy	810.86 ± 844.24	812.50 ± 828.78	1188.36 ± 1014.46	1.000	0.036	0.012
	Paired t test/p-value^#^	0.009	0.258	0.001			
Sweetened teas**	Pre-pregnancy	1.99 ± 1.82	2.71 ± 2.11	3.96 ± 1.86	0.029	< 0.001	< 0.001
	Early pregnancy	1.85 ± 1.65	2.53 ± 2.06	3.30 ± 2.25	0.043	0.048	< 0.001
	Paired t test/p-value^#^	0.285	0.480	0.008	–		
Carbonated drinks**	Pre-pregnancy	0.51 ± 1.03	0.37 ± 0.69	0.60 ± 1.02	0.559	0.327	0.805
	Early pregnancy	0.33 ± 0.64	0.18 ± 0.48	0.38 ± 0.64	0.234	0.132	0.817
	Paired t test/p-value^#^	0.285	0.150	0.035	–		
Coffee**	Pre-pregnancy	1.76 ± 2.30	1.89 ± 2.30	1.90 ± 2.15	0.918	1.000	0.909
	Early pregnancy	0.60 ± 1.34	0.57 ± 1.26	0.59 ± 1.20	0.980	0.994	0.997
	Paired t test/p-value^#^	< 0.001	< 0.001	< 0.001	–		
Juice**	Pre-pregnancy	0.72 ± 1.20	0.68 ± 1.19	0.79 ± 1.17	0.981	0.853	0.901
	Early pregnancy	0.55 ± 0.89	0.49 ± 0.74	0.63 ± 0.99	0.892	0.612	0.799
	Paired t test/p-value^#^	0.135	0.140	0.128	–		
Water^&^	Pre-pregnancy	973.68 ± 303.34	930.92 ± 323.00	791.10 ± 353.59	0.638	0.031	< 0.001
	Early pregnancy	1008.22 ± 291.18	986.84 ± 310.63	815.07 ± 353.62	0.888	0.004	< 0.001
	Paired t test/p-value^#^	0.068	0.026	0.490	–		

Numbers are given in mean ± standard deviations (SD).

*One-way ANOVA was used to analyze low and mild modified DSM-5-TR score groups.

^One-way ANOVA was used to analyze mild and moderate + modified DSM-5-TR score groups.

^@^One-way ANOVA was used to analyze low and moderate + modified DSM-5-TR score groups.

^#^Paired-t test was used to analyze pre-pregnancy and early pregnancy changes, p < 0.05 denotes statistical significance.

^&^Variables measured in mL per day.

**Variables measured in times per week.

**Figure 3:**
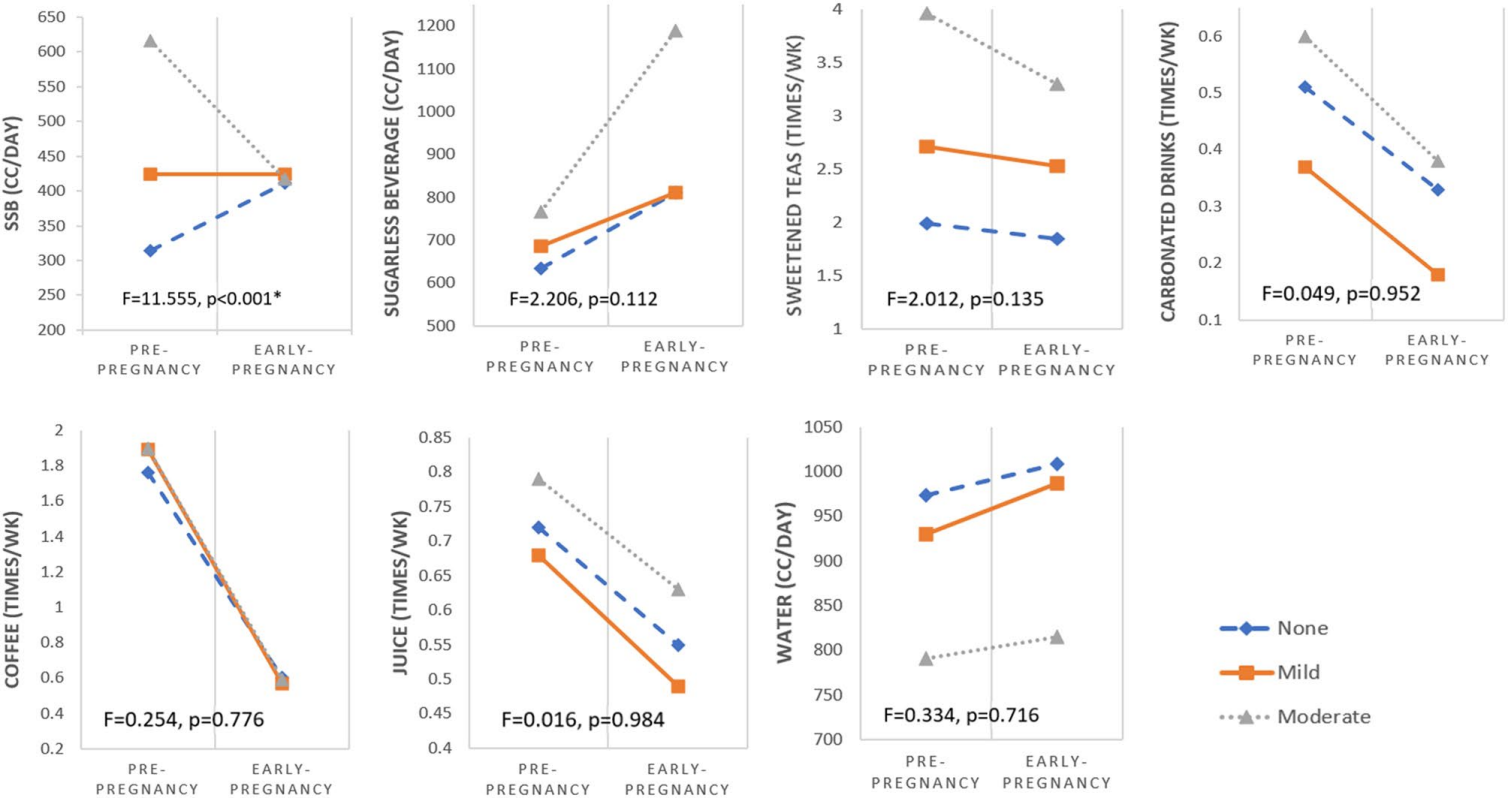
Beverage consumption patterns before and in early pregnancy sorted by modified DSM-5-TR scores of low, mild and moderate+ (adjusted by age), using general linear model for repeated measurement analysis. *Denotes statistical significance, p < 0.05.

Significant changes of beverage choices during the pre- to early-pregnancy transition, both in amount and direction, were presented in Table 2, Figs. 1C and 3. Increased consumption in sugarless beverage was uniformly observed (p = 0.009, p = 0.258, p = 0.001 for the low, mild and moderate + DSM-5-TR groups, respectively). Decreased intake were most profound in sweetened teas (p = 0.008), carbonated drinks (p = 0.035) and coffee (p < 0.001), especially in the moderate + groups. SSB has different change patterns in different DSM-5-TR score groups during this transition. Those scored low increased their SSB consumption after becoming pregnant with statistical significance (314 mL/day to 412 mL/day, p = 0.003); while those scored moderated + in DSM-5-TR scores decreased their SSB intake (616 mL/day to 417 mL/day, p < 0.001).

## Discussion

Pregnant women’s inclination for SSB differed by their DSM-5-TR scores. Participants with higher DSM-5 score before pregnancy reduced SSB consumption after becoming pregnant; those with low DSM-5 scores consumed significantly more after the status transition (Fig. 1C). It is common for women to actively change their diet and seek nutrition supplements such as minerals and vitamins to optimize their fetal development. Similarly, some women perceive free sugar as source for energy and fetal growth so the low scorers might increase their SSB intake after becoming pregnant. The results of cutting carbonated drinks, caffeine and sweetened teas, while increasing sugarless beverage and plain water are common. The only other published work that shared similar study objective was from Skreden et al., examining Norwegian nulliparous women’s diet changes in early pregnancy, with mirroring results^33^. Of note, women with high DSM-5-TR scores reduced SSB consumption but shifted largely to sugarless beverage in contrast to the relatively stable amount of plain water. While artificially sweeteners are common substitutes for SSB, they also demonstrated adverse health outcomes for the maternal fetal subjects^42^. Pregnant women should be reminded and encouraged to drink more plain water rather than sugarless beverages.

Although SSB as an SUD is not validated and largely controversial, its characterization using modified DSM-5-TR SUD criteria provides valuable insights for the psychometric factors in the consumption process. Clinicians and patients alike could be made more aware of the factors underscoring beverage consumption to make practical suggestions. According to our data, participants responded the most in “SSB consumption despite of hazardous health effect”. This indicated that reproductive women were already equipped with basic knowledge of healthy beverage choice during pregnancy. Instructions, messages pertaining to healthy diet were readily available through social media and public education, but these were not enough to deter them. One potential explanation is that the physiologic consequences from SSB on pregnant women is often delayed or too subtle. Obesity, cardiovascular diseases, metabolic syndrome, or even fetal outcomes would not be apparent months, years or even decades later. This contrasts with the alarming symptoms such as hallucinations, chills, shakes, pupil dilation, incontinence, caused by other substances that illicit immediate attention. The delayed consequences should be stressed.

On the other hand, effect of SSB on social networking or work performance were unremarkable (Fig. 2). Previous studies exploring the impact of peers on unhealthy eating are mostly based on adolescent populations. Most agreed that peers played a role in SSB consumption and programs aiming to reduce SSB intake should involve peers^43–46^. On the contrary, the present study on pregnant women did not observe an impact on social networking or peers by SSB consumption. This could be explained by the ease of access of SSB in Taiwan. Taiwan ranked second highest in the world for ratio of convenience stores per population since 2018^47^. 2020 statistics released by the Fair Trade Commission of Taiwan reported that Taiwanese visit convenience stores 137 times per year annually and spend 84 New Taiwanese dollars per visit^48^. SSB is so easy to acquire both in price and distribution that women could consume them without affecting their interpersonal relationships or work. The normalization of SSB in Taiwan culture may have skewed the results towards no effect, but this should not change what the data concludes because it shifted the overall DSM-5-TR scores in one direction. Also, scores for tolerance (n = 24) and withdrawal presentations (n = 70) were not so pronounced. Since animals rely on sugar as energy source since the beginning of time, it is unsurprising that there is high tolerance for it. This revealed that SSB consumption in pregnancy is unlikely a result of pharmacological effect induced by addictive substances, as do by opioids, cocaine and nicotine^24,49,50^. We then have to rely on impairment or other categories to explain drinker behavior. Additionally, it might be more effective for an obstetrician to emphasize impaired control and risky use when consulting their pregnant women in cutting SSB consumption. The relationship between SSB and PPD is more likely a result of direct biological effects via alterations in hypothalamic–pituitary–adrenal axis^51^ or gut bacteria mediated memory functions^52^ (Fig. 1A). It warrants future studies for modified SUD test to exclude these no-applicable categories to obtain more accurate data.

Our work is the first to characterize SSB consumption in pregnant with modified DSM-5-TR SUD scores with repeated measurements before and in early pregnancy. The research setting is a tertiary teaching hospital that complies with national health insurance, covering patients from all socioeconomic backgrounds. With unselective sequential invitations and high response rate, the sample capture bias is limited. The score correlated well with SSB consumption amount, and the changed patterns of the various beverage types agreed with previously published works. Both provided validation for the utilization of DSM-5-TR SUD diagnostic criteria in our study objective. Data acquired from such framework allows us to analyze and design effective strategies that target the underlying causes of SSB consumption by women in early pregnancy.

There are some limitations to report. The self-report questionnaire is prone for recall bias and misreporting, especially when responders tend to underreport food they perceived as unhealthy to a greater extent than food perceived as healthy^53,54^. The total diet including vegetables, fruits, grains, vitamins, and other sources of sugars were not accounted for. The category “sugarless beverages” was a collective term that included artificially sweetened beverages, unsweetened ice tea, unsweetened lemonades…etc., and their individual effects to DSM-5-TR symptoms were not examined. Finally, the application of DSM-5-TR SUD criteria in SSB consumption is not validated. Our study objective was not to make disease diagnosis, but rather to depict the underlying psychomotor elements leading to beverage drinking behaviors. DSM-5-TR diagnostic criteria design has the flexibility and acceptability of such utilization in the research settings^21,27,29,55^.

Previous studies reported decreased SSB intake by pregnant women collectively as a cohort. The current study examined these women’s psychometric perceptions towards SSB. Those reported moderate or high DSM-5-TR scores reduced SSB intake after becoming pregnant. Their choice of liquid intake largely shifted to sugarless drinks and to a lesser extent plain water, which should be reminded and encouraged more. Those claimed low DSM-5-TR scores consumed more SSB after becoming pregnant. The most significant factor was having risky use and impaired control despite acknowledging adverse health consequences. The least reported factor was in the domain of social impairment. The unique feature of characterizing SSB consumption in early pregnancy by a DSM-5-TR based tool provided new insight of the underlying psychometric factors of these women. The role of SSB as an addictive substance was not indicated by our analysis. Clinicians and patients alike should be made more aware of the SSB consumption patterns and giving counter strategies accordingly. The results added to our understandings of SSB intake by women in early pregnancy and provided useful reference for making clinical recommendations.

## Methods

This longitudinal observational cohort study was conducted at Kaohsiung Medical University Hospital (KMUH). The project was approved by the Institutional Review Board of Kaohsiung Medical University Hospital (KMUHIRB-SV(I)-20160062) and executed in accordance with the Declaration of Helsinki. Since August 2018, we invited all pregnant women visiting the clinic to answer a structured 20-min questionnaire every trimester during the regular antennal visit. At the end of the study, 337 pregnant women aged over 20 participated. After obtaining their written informed consents, the questionnaire assessed participants’ basic demographic information, biometric measurements at study entry, obstetric history, diet style, beverage drinking patterns and exercise routines for the past 3 months. The participants received their first questionnaire in the first prenatal visit, which assessed their beverage consumption in the past 3 months or preconceptionally. The same questionnaire was answered in the second trimester that assessed beverage consumption patterns in their first trimester. Those who declined the offer, were unable read Mandarin Chinese, failed to complete all 3 questionnaires, had unexpected termination of pregnancy, intrauterine fetal demise or switched antenatal care elsewhere were excluded. A total of 301 responses entered for final analysis, with a response rate of 89.3%.

Participants’ beverages drinking patterns were depicted by types (sugar sweetened drinks, sugarless drinks, carbonated beverages, sweetened teas, coffee, juice, water), frequency of use (times per week) or estimated intake amount (mL/day). The diagnostic criteria of SUD in DSM-5-TR were modified to 11 beverage-specific questions to assess the participants’ socio-psychomotor status in the dimensions of impaired control, social impairment, risky use and pharmacologic symptoms towards these drinks^21^. The numbers of responses to the modified DSM-5-TR questions were grouped into low (0–1 point), mild (2–3 points) and moderate plus (≥ 4 points) groups for analysis.

Statistical studies were performed using SPSS v20.0.0 (International Business Machines Corporation, College Station, New York, United States of America). Demographic information of the study cohort and modified DSM-5-TR scores were given in proportions and means ± standard deviations (SD). To reduce residual confounding effect, multivariable models were adjusted for age, educational level, body mass index and nulliparity. One-way analysis of variance (ANOVA) was used to examine differences between DSM-5-TR score groups (low versus mild, mild versus moderate + and low versus moderate +). Paired t-test was used to depict differential pre-pregnancy and early pregnancy drinking patterns. The dynamic trends of the diverse types of beverages consumed before-after pregnancy by means of DSM-5-TR scores were demonstrated. The significance of these changes were measured by general linear model for repeated measure analysis. Statistically significant results were given in P-values less than 0.05.

### Supplementary Information

Supplementary Table 1.

## Data Availability

The datasets used and analyzed in the current study are available from the corresponding author upon reasonable request.
