# Supplementary material for: Assessing sugar-sweetened beverage consumption in early pregnancy using a substance abuse framework
Source: Sci Rep. 2023 Nov 3;13:18979. doi: 10.1038/s41598-023-46265-y (PMC10624895; doi:10.1038/s41598-023-46265-y)
Supplement: Supplementary file 1 — Supplementary Table 1. [file 41598_2023_46265_MOESM1_ESM.docx]

| Groups based on DSM-5 scores* 🡪 | | Low (n=152) | | Mild (n=76) | | Moderate + (n=73) | | | Sum |
| --- | --- | --- | --- | --- | --- | --- | --- | --- | --- |
| Number of responses 🡪 | | 0 (n=110) | 1 (n=42) | 2 (n=36) | 3 (n=40) | 4 (n=28) | 5 (n=20) | 6+ (n=25) | (n=301) |
| Impaired control | D1. Increased consumption | 0 | 4 | 5 | 7 | 9 | 6 | 13 | 44 |
|  |  | 9.0% | | 27.2% | | 63.6% | | |  |
|  | D2. Increased desire | 0 | 4 | 11 | 18 | 18 | 14 | 23 | 88 |
|  |  | 4.5% | | 33% | | 62.5% | | |  |
|  | D3. Excessive time expenditure | 0 | 3 | 8 | 17 | 19 | 15 | 23 | 85 |
|  |  | 3.5% | | 29.4% | | 67.0% | | |  |
|  | D4. Restless until consumption | 0 | 6 | 9 | 12 | 15 | 16 | 23 | 81 |
|  |  | 7.4% | | 25.9% | | 66.7% | | |  |
| Social impairment | D5. Affected work performance | 0 | 0 | 1 | 0 | 1 | 2 | 3 | 7 |
|  |  | 0.0% | | 33.3% | | 66.7% | | |  |
|  | D6. Affected relationships | 0 | 1 | 1 | 2 | 3 | 1 | 18 | 26 |
|  |  | 3.8% | | 11.5% | | 84.6% | | |  |
|  | D7. Reduced social networking | 0 | 1 | 0 | 0 | 0 | 0 | 2 | 3 |
|  |  | 33.3% | | 0.0% | | 66.7% | | |  |
| Risky use | D8. Despite of bodyweight gain | 0 | 3 | 6 | 17 | 12 | 10 | 15 | 63 |
|  |  | 4.8% | | 36.5% | | 58.7% | | |  |
|  | D9. Despite of known hazards | 0 | 19 | 18 | 28 | 24 | 19 | 25 | 133 |
|  |  | 14.3% | | 34.6% | | 51.1% | | |  |
| Pharmacologic symptoms | D10. Tolerance presentation | 0 | 0 | 1 | 4 | 1 | 5 | 13 | 24 |
|  |  | 0.0% | | 20.8% | | 79.1% | | |  |
|  | D11 Withdrawal presentation | 0 | 1 | 12 | 15 | 10 | 12 | 20 | 70 |
|  |  | 1.4% | | 38.6% | | 57.5% | | |  |
| Range of positive responses 🡪 | | 0.0-12.5% | | 0.0-60.5% | | 2.7-93.2% | | | ---------- |

Supplementary Table 1. Raw data of participants’ responses sorted by degree of beverage use scores (low, mild, moderate+) and specific DSM-5 questions (D1-D11). Numbers are given in number(n) or percentage (%). * Each positive response scores one point. Degree of SSB use awareness is classified by points scored: low (0-1 point), mild (2-3 points) and moderate + (≥ 4 points)
